# 2-Amino-6-methyl­pyridinium 4-methyl­benzene­sulfonate

**DOI:** 10.1107/S1600536814008587

**Published:** 2014-04-26

**Authors:** K. Syed Suresh Babu, M. Dhavamurthy, M. NizamMohideen, G. Peramaiyan, R. Mohan

**Affiliations:** a Department of Physics, Presidency College (Autonomous), Chennai 600 005, Tamil Nadu, India; b Department of Physics, The New College (Autonomous), Chennai 600 014, Tamil Nadu, India

## Abstract

In the asymmetric unit of the title salt, C_6_H_9_N_2_^+^·C_7_H_7_O_3_S^−^, there are two independent 2-amino-6-methyl­pyridinium cations and two independent 4-methyl­benzene­sulfonate anions. Both cations are protonated at their pyridine N atoms and their geometries reveal amine–imine tautomerism. In the 4-methyl­benzene­sulfonate anions, the carboxyl­ate groups are twisted out of the benzene ring planes by 88.4 (1) and 86.2 (2)°. In the crystal, the sulfonate O atoms of an anion inter­act with the protonated N atoms and the 2-amino groups of a cation *via* a pair of N—H⋯O hydrogen bonds, forming an *R*_2_^2^(8) ring motif. These motifs are connected *via* N—H⋯O hydrogen bonds, forming chains running along the *a-*axis direction. Within the chains there are weak C—H⋯O hydrogen bonds present. In addition, aromatic π–π stacking inter­actions [centroid–centroid distances = 3.771 (2), 3.599 (2), 3.599 (2) and 3.497 (2) Å] involving neighbouring chains are also observed.

## Related literature   

For crystal structures of related pyridine derivatives and their applications, see: Babu *et al.* (2014 ▶); Rajkumar *et al.* (2014 ▶); Jin *et al.* (2005 ▶). For unprotonated amino­pyridine derivatives, see: Anderson *et al.* (2005 ▶). For the structure of amino-methyl­pyridinium, see: Nahringbauer & Kvick (1977 ▶). For details of sulfonates, see: Onoda *et al.* (2001 ▶); Baskar Raj *et al.* (2003 ▶). For applications of benzene­sulfonic acid, see: Wang & Wei (2007 ▶). For simple organic–inorganic salts containing strong inter­molecular hydrogen bonds, see: Sethuram *et al.* (2013*a* ▶,*b* ▶); Shihabuddeen Syed *et al.* (2013 ▶); Showrilu *et al.* (2013 ▶); Huq *et al.* (2013 ▶). For bond-length data, see: Allen *et al.* (1987 ▶). For studies on the tautomeric forms of 2-amino­pyridine systems, see: Ishikawa *et al.* (2002 ▶). For graph-set analysis, see: Etter (1990 ▶); Bernstein *et al.* (1995 ▶).
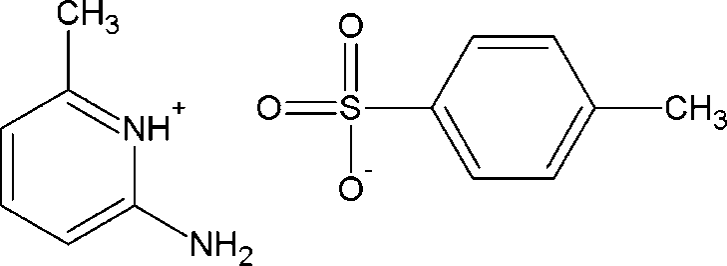


## Experimental   

### 

#### Crystal data  

C_6_H_9_N_2_^+^·C_7_H_7_O_3_S^−^
*M* *_r_* = 280.35
Triclinic, 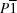

*a* = 7.5343 (2) Å
*b* = 13.6212 (5) Å
*c* = 13.9887 (5) Å
α = 106.307 (2)°
β = 97.946 (1)°
γ = 92.103 (2)°
*V* = 1360.31 (8) Å^3^
*Z* = 4
Mo *K*α radiation
μ = 0.24 mm^−1^
*T* = 293 K
0.35 × 0.25 × 0.20 mm

#### Data collection  

Bruker Kappa APEXII CCD diffractometer
Absorption correction: multi-scan (*SADABS*; Sheldrick, 2004 ▶) *T*_min_ = 0.920, *T*_max_ = 0.953
32534 measured reflections
6237 independent reflections
4709 reflections with *I* > 2σ(*I*)
*R*_int_ = 0.026

#### Refinement  

*R*[*F* ^2^ > 2σ(*F* ^2^)] = 0.040
*wR*(*F* ^2^) = 0.119
*S* = 1.06
6237 reflections
372 parameters
6 restraints
H atoms treated by a mixture of independent and constrained refinement
Δρ_max_ = 0.33 e Å^−3^
Δρ_min_ = −0.37 e Å^−3^

### 

Data collection: *APEX2* (Bruker, 2004 ▶); cell refinement: *APEX2* and *SAINT* (Bruker, 2004 ▶); data reduction: *SAINT* and *XPREP* (Bruker, 2004 ▶); program(s) used to solve structure: *SHELXS97* (Sheldrick, 2008 ▶); program(s) used to refine structure: *SHELXL97* (Sheldrick, 2008 ▶); molecular graphics: *ORTEP-3 for Windows* (Farrugia, 2012 ▶) and *Mercury* (Macrae *et al.*, 2008 ▶); software used to prepare material for publication: *WinGX* (Farrugia, 2012 ▶) and *PLATON* (Spek, 2009 ▶).

## Supplementary Material

Crystal structure: contains datablock(s) global, I. DOI: 10.1107/S1600536814008587/su2726sup1.cif

Structure factors: contains datablock(s) I. DOI: 10.1107/S1600536814008587/su2726Isup2.hkl

Supporting information file. DOI: 10.1107/S1600536814008587/su2726Isup3.cml

CCDC reference: 997539

Additional supporting information:  crystallographic information; 3D view; checkCIF report

## Figures and Tables

**Table 1:** Hydrogen-bond geometry (Å, °)

*D*—H⋯*A*	*D*—H	H⋯*A*	*D*⋯*A*	*D*—H⋯*A*
N1—H1*A*⋯O2	0.90 (1)	1.88 (1)	2.772 (2)	171 (2)
N2—H2*A*⋯O3^i^	0.87 (1)	2.01 (1)	2.880 (2)	174 (2)
N2—H2*B*⋯O1	0.88 (1)	2.07 (1)	2.919 (2)	162 (2)
N3—H3*A*⋯O5	0.89 (1)	1.90 (1)	2.789 (2)	174 (2)
N4—H4*A*⋯O4^ii^	0.88 (1)	2.02 (1)	2.882 (2)	167 (2)
N4—H4*B*⋯O6	0.88 (1)	2.04 (1)	2.883 (2)	162 (2)
C22—H22⋯O5^ii^	0.93	2.58	3.455 (2)	157

Symmetry codes: (i) 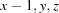; (ii) 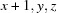.

**Fig. 1.**
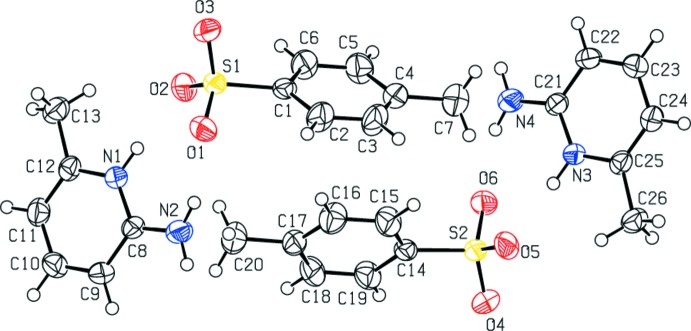
A view of the molecular structure of the two independent benezesulfonate anions and the two independent 2-amino-6-methylpyridinium cations of the title salt. Displacement ellipsoids are drawn at the 50% probability level.

**Fig. 2.**
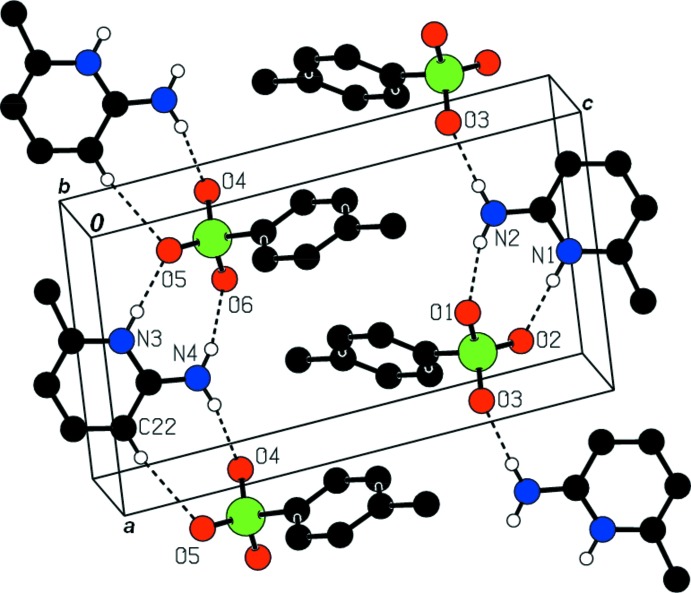
The crystal packing of the title compound, viewed along the b axis. The N—H···O and C—H···O hydrogen bonds are shown as dashed lines (see Table 1 for details; H atoms not involved in hydrogen bonding have been omitted for clarity).

## supplementary crystallographic information

### 1. Introduction

2-Amino­pyridine and its derivatives play an important role in heterocyclic chemistry. Pyridine heterocycles and their derivatives are present in many large molecules having photo-chemical, electro-chemical and catalytic applications (Babu *et al.*, 2014). Simple organic-inorganic salts containing strong inter­molecular hydrogen bonds have attracted an attention as materials which display ferroelectric-paraelectric phase transitions (Sethuram, *et al.*, 2013*a*,*b*; Huq *et al.*, 2013; Shihabuddeen Syed *et al.*, 2013; Showrilu *et al.*, 2013). Hydrogen-bonding patterns involving sulfonate groups in biological systems and metal complexes are of current inter­est (Onoda *et al.*, 2001). Such inter­actions can be utilized for designing supra­molecular architectures (Baskar Raj *et al.*, 2003). Benzene­sulfonic acid, is a particularly strong organic acid which is capable of protonating N-containing heterocycles and other Lewis bases (Wang & Wei, 2007). We have recently reported the crystal structures of 2-amino-6-methyl­pyridinium 2,2,2-tri­chloro­acetate (Babu *et al.*, 2014) and 2-Amino-5-nitro­pyridinium hydrogen oxalate (Rajkumar *et al.*, 2014). In continuation of our studies of pyridinium derivatives, the crystal structure determination of the title compound has been undertaken.

### 2. Comment / Result and Discussion

The asymmetric unit of title salt, Fig. 1, consists of two crystallographically independent protonated 2-amino-6-methyl­pyridinium cation and two crystallographically independent 4-methyl benzene­sulfonate anions. The normal probability plot analyses (Inter­national Tables for X-ray Crystallography, 1974, Vol. IV, pp. 293–309) for both bond lengths and angles show that the differences between the two symmetry independent molecules are of a statistical nature. All bond lengths (Allen *et al.*, 1987) and angles are within normal ranges and comparable with those in closely related structures (Babu *et al.*, 2014; Rajkumar *et al.*, 2014). A proton transfer from the carboxyl group of *p*-toluene­sulfonic acid to atom N1 and N3 of 2-amino-6-methyl pyridine resulted in the formation of a salt. This protonation lead to the widening of the C8—N1—C12 and C21—N3—C25 angles of the pyridine rings to 124.0 (2) ° and 123.8 (2) °, compared to 115.3 (2) ° in the unprotonated amino­pyridine (Anderson *et al.*, 2005). This type of protonation is observed in various amino­pyridine acid complexes (Babu *et al.*, 2014; Rajkumar *et al.*, 2014).

In the cation, the N2—C8 [1.325 (2) Å] N4—C21 bonds [1.325 (2) Å] is shorter than the N1—C8 [1.347 (2) Å], N1—C12 [1.360 (2) Å], N3—C21[1.352 (2) Å] and N3—C25[1.362 (2) Å] bonds, and the C8—C9 [1.406 (3) Å], C10—C11 [1.398 (3) Å], C21—C22 [1.405 (3) Å] and C23—C24 [1.401 (3) Å] bonds are significantly longer than C9—C10 [1.357 (3) Å], C11—C12 [1.356 (3) Å]. C22—C23 [1.357 (3) Å] and C24—C25 [1.353 (3) Å] bonds, are similar to those in the amino-methyl­prydinium cation (Babu *et al.*, 2014; Rajkumar *et al.*, 2014). In contrast, in the solid state structure of amino-methyl­pyridinium, the N—C bond out of ring is clearly longer than that in the ring (Nahringbauer *et al.*, 1977). The geometrical features of the amino-methyl­pyridinium cation (N1/N2/C1/C6 and N3/N4/C9—C13) resemble those observed in other 2-amino­pyridinium structures (Babu *et al.*, 2014; Rajkumar *et al.*, 2014) that are believed to be involved in amine-imine tautomerism (Ishikawa *et al.*, 2002). Similar features are also provided by cation amino-methyl­pyridinium (N3/N4/C7/C12). However, previous study show that a pyridinium cation always possesses an expanded angle of C—N—C in comparison with the parent pyridine (Jin *et al.*, 2005).

The examination of pyridinium rings shows that these units are planar with mean deviation of -0.006 (2) and 0.005 (2) Å for atoms C8 and C21, from the mean planes defined by the six constituent atoms. The dihedral angle between the 2-amino-6-methyl­pyridinium cation and 4-methyl­benzene­sulfonate anion group is 88.4 (2) and 86.2 (2)° for the both molecules, respectively. In both the molecules, the protonated 2-amino-6-methyl­pyridinium cation is essentially planar, with maximum deviations of -0.012 (2) for atom C13 and -0.006 (2) Å for atom C25.

### 3. Hydrogen bonding inter­action / Inter­molecular N—H···O and C—H···O inter­action

In the crystal (Fig. 2), the protonated atoms (N1 and N3) and a nitro­gen atom of the 2-amino groups (N2 and N4) of the 2-amino-6-methyl­pyridinium cations are hydrogen bonded to the carboxyl­ate oxygen atoms (O1, O2, O3 and O4) of the sulfonate groups of the *p*-toluene­sulfonate anions *via* a pair of inter­molecular N—H···O hydrogen bonds (Table 1), forming a ring motif with a graph-set notation of *R*^2^_2_(8) [Etter, 1990; Bernstein *et al.*, 1995]. The sulfonate group mimics the carboxyl­ate anion's mode of association, which is more commonly seen when binding with 2-amino­pyrimidines. It is well known that sulfonates imitate carboxyl­ates in forming such bidentate motifs (Baskar Raj *et al.*, 2003).

Furthermore, these motifs are connected *via* N—H···O hydrogen bonds (Fig. 2 and Table 1), involving the 2-amino group of the 2-amino-6-methyl pyridinium cation and atoms O3 and O4 of an anion, to form a supra­molecular chains along the a axis direction. Weak C—H···O hydrogen bonds, involving a pyridine group of the cation and an O atom of a sulfonate anion, within the chains are also observed (Fig. 2 and Table 1).

### 4. Aromatic inter­action

In addition, the cations of neighbouring chains are linked through aromatic π-π inter­actions with centroid distances *Cg*1···*Cg*1^iii^ = 3.771 (2), *Cg*1···*Cg*2^iv^ = 3.599 (2), *Cg*2···*Cg*1^v^ = 3.599 (2) and *Cg*2···*Cg*2^vi^ = 3.497 (2) Å [symmetry codes are as in Table 1 and (iii) = -x*+1*,-y+1,-z+2; (iv) = x, y, z+ (v) = x, y, z+1; (vi) = -x+1, -y, -z; *Cg*1 and *Cg*2 are the centroids of the N1/C8—C12 and N3/C21—C25 rings, respectively].

### 5. Uses

The identification of such supra­molecular patterns will help us design and construct preferred hydrogen bonding patterns of drug like molecules.

### 6. Synthesis and crystallization

Crystals of the title compound were obtained by slow evaporation of a 1:1 equimolar mixture of 2-amino-6-methyl­pyridine and benzene­sulfonic acid in methanol at room temperature.

### 7. Refinement

Crystal data, data collection and structure refinement details are summarized in Table 1. N-bound H atoms were located in a difference Fourier map and refined with distance restraints: N—H = 0.88 (1) and 0.90 (1) Å for NH_2_ and NH H atoms, respectively. The C-bound H atoms were positioned geometrically and refined using a riding model: C—H = 0.93–0.96 Å with *U*_iso_(H) = 1.5*U*_eq_(*C*-methyl) and = 1.2*U*_eq_(C) for other H atoms. A rotating group model was used for the methyl group.

### Crystal data

**Table:**

C_6_H_9_N_2_^+^·C_7_H_7_O_3_S^−^	*Z* = 4
*M**_r_* = 280.35	*F*(000) = 592
Triclinic, *P*1	*D*_x_ = 1.369 Mg m^−^^3^
Hall symbol: -P 1	Mo *K*α radiation, λ = 0.71073 Å
*a* = 7.5343 (2) Å	Cell parameters from 6237 reflections
*b* = 13.6212 (5) Å	θ = 2.0–28.1°
*c* = 13.9887 (5) Å	µ = 0.24 mm^−^^1^
α = 106.307 (2)°	*T* = 293 K
β = 97.946 (1)°	Block, colourless
γ = 92.103 (2)°	0.35 × 0.25 × 0.20 mm
*V* = 1360.31 (8) Å^3^	

### Data collection

**Table:**

Bruker Kappa APEXII CCD diffractometer	6237 independent reflections
Radiation source: fine-focus sealed tube	4709 reflections with *I* > 2σ(*I*)
Graphite monochromator	*R*_int_ = 0.026
ω and φ scans	θ_max_ = 27.5°, θ_min_ = 1.6°
Absorption correction: multi-scan (*SADABS*; Sheldrick, 2004)	*h* = −9→9
*T*_min_ = 0.920, *T*_max_ = 0.953	*k* = −17→17
32534 measured reflections	*l* = −18→18

### Refinement

**Table:**

Refinement on *F*^2^	Secondary atom site location: difference Fourier map
Least-squares matrix: full	Hydrogen site location: inferred from neighbouring sites
*R*[*F*^2^ > 2σ(*F*^2^)] = 0.040	H atoms treated by a mixture of independent and constrained refinement
*wR*(*F*^2^) = 0.119	*w* = 1/[σ^2^(*F*_o_^2^) + (0.0502*P*)^2^ + 0.655*P*] where *P* = (*F*_o_^2^ + 2*F*_c_^2^)/3
*S* = 1.06	(Δ/σ)_max_ = 0.001
6237 reflections	Δρ_max_ = 0.33 e Å^−^^3^
372 parameters	Δρ_min_ = −0.37 e Å^−^^3^
6 restraints	Extinction correction: *SHELXL97* (Sheldrick, 2008), Fc^*^=kFc[1+0.001xFc^2^λ^3^/sin(2θ)]^-1/4^
Primary atom site location: structure-invariant direct methods	Extinction coefficient: 0.0067 (10)

### Special details

**Table:**

Geometry. All e.s.d.'s (except the e.s.d. in the dihedral angle between two l.s. planes) are estimated using the full covariance matrix. The cell e.s.d.'s are taken into account individually in the estimation of e.s.d.'s in distances, angles and torsion angles; correlations between e.s.d.'s in cell parameters are only used when they are defined by crystal symmetry. An approximate (isotropic) treatment of cell e.s.d.'s is used for estimating e.s.d.'s involving l.s. planes.
Refinement. Refinement of *F*^2^ against ALL reflections. The weighted *R*-factor *wR* and goodness of fit *S* are based on *F*^2^, conventional *R*-factors *R* are based on *F*, with *F* set to zero for negative *F*^2^. The threshold expression of *F*^2^ > σ(*F*^2^) is used only for calculating *R*-factors(gt) *etc*. and is not relevant to the choice of reflections for refinement. *R*-factors based on *F*^2^ are statistically about twice as large as those based on *F*, and *R*- factors based on ALL data will be even larger.

### Fractional atomic coordinates and isotropic or equivalent isotropic displacement parameters (Å^2^)

**Table:**

	*x*	*y*	*z*	*U*_iso_*/*U*_eq_	
C1	0.7573 (2)	0.33148 (14)	0.65370 (12)	0.0340 (4)	
C2	0.6441 (3)	0.28156 (17)	0.56563 (15)	0.0510 (5)	
H2	0.5822	0.2192	0.5594	0.061*	
C3	0.6232 (3)	0.32433 (19)	0.48708 (16)	0.0568 (6)	
H3	0.5472	0.2897	0.4279	0.068*	
C4	0.7111 (3)	0.41650 (17)	0.49332 (15)	0.0450 (5)	
C5	0.8219 (3)	0.46630 (18)	0.58213 (16)	0.0525 (5)	
H5	0.8818	0.5293	0.5886	0.063*	
C6	0.8455 (3)	0.42432 (17)	0.66170 (15)	0.0498 (5)	
H6	0.9213	0.4590	0.7209	0.060*	
C7	0.6848 (3)	0.4621 (2)	0.40658 (18)	0.0658 (7)	
H7A	0.7653	0.4341	0.3602	0.099*	
H7B	0.7093	0.5352	0.4315	0.099*	
H7C	0.5630	0.4461	0.3727	0.099*	
O1	0.63184 (18)	0.20570 (11)	0.73960 (10)	0.0473 (3)	
O2	0.80180 (19)	0.36019 (11)	0.84663 (10)	0.0482 (3)	
O3	0.95409 (17)	0.22525 (12)	0.74752 (10)	0.0487 (4)	
S1	0.78890 (6)	0.27601 (4)	0.75373 (3)	0.03657 (13)	
C14	0.1612 (2)	0.16545 (14)	0.35185 (12)	0.0335 (4)	
C15	0.2427 (3)	0.07546 (17)	0.34413 (15)	0.0497 (5)	
H15	0.2824	0.0416	0.2843	0.060*	
C16	0.2656 (3)	0.03531 (17)	0.42480 (16)	0.0532 (5)	
H16	0.3205	−0.0258	0.4186	0.064*	
C17	0.2085 (3)	0.08433 (16)	0.51461 (14)	0.0427 (4)	
C18	0.1255 (3)	0.17361 (18)	0.52060 (15)	0.0522 (5)	
H18	0.0851	0.2074	0.5802	0.063*	
C19	0.1007 (3)	0.21434 (16)	0.44031 (15)	0.0471 (5)	
H19	0.0434	0.2746	0.4459	0.057*	
C20	0.2366 (3)	0.0415 (2)	0.60338 (17)	0.0610 (6)	
H20A	0.3489	0.0705	0.6450	0.092*	
H20B	0.2384	−0.0317	0.5797	0.092*	
H20C	0.1403	0.0584	0.6420	0.092*	
O4	−0.01417 (17)	0.27792 (12)	0.25616 (11)	0.0496 (4)	
O5	0.13593 (18)	0.13852 (11)	0.15948 (9)	0.0471 (3)	
O6	0.30945 (17)	0.28838 (11)	0.27035 (10)	0.0453 (3)	
S2	0.14592 (6)	0.22210 (4)	0.25238 (3)	0.03629 (13)	
C21	0.5816 (2)	0.16145 (13)	0.08860 (13)	0.0327 (4)	
C22	0.7160 (2)	0.14424 (14)	0.02675 (14)	0.0368 (4)	
H22	0.8366	0.1588	0.0550	0.044*	
C23	0.6682 (2)	0.10619 (15)	−0.07461 (14)	0.0407 (4)	
H23	0.7569	0.0954	−0.1157	0.049*	
C24	0.4871 (2)	0.08288 (14)	−0.11818 (14)	0.0392 (4)	
H24	0.4556	0.0568	−0.1877	0.047*	
C25	0.3584 (2)	0.09863 (13)	−0.05818 (13)	0.0340 (4)	
C26	0.1613 (2)	0.07772 (16)	−0.09541 (15)	0.0452 (5)	
H26A	0.1106	0.1403	−0.0996	0.068*	
H26B	0.1047	0.0509	−0.0496	0.068*	
H26C	0.1419	0.0285	−0.1609	0.068*	
N3	0.40875 (19)	0.13737 (11)	0.04333 (11)	0.0325 (3)	
N4	0.6161 (2)	0.19844 (15)	0.18808 (12)	0.0445 (4)	
C8	0.3602 (2)	0.33977 (13)	0.91967 (13)	0.0334 (4)	
C9	0.2264 (2)	0.35815 (15)	0.98204 (14)	0.0393 (4)	
H9	0.1055	0.3440	0.9543	0.047*	
C10	0.2761 (3)	0.39686 (16)	1.08322 (15)	0.0449 (5)	
H10	0.1883	0.4085	1.1248	0.054*	
C11	0.4573 (3)	0.41950 (16)	1.12590 (14)	0.0432 (4)	
H11	0.4896	0.4463	1.1953	0.052*	
C12	0.5854 (2)	0.40210 (13)	1.06520 (13)	0.0348 (4)	
C13	0.7826 (3)	0.42082 (16)	1.10162 (15)	0.0460 (5)	
H13A	0.8040	0.4713	1.1664	0.069*	
H13B	0.8402	0.4453	1.0547	0.069*	
H13C	0.8305	0.3580	1.1072	0.069*	
N1	0.53289 (19)	0.36343 (11)	0.96397 (11)	0.0326 (3)	
N2	0.3236 (2)	0.30260 (14)	0.82019 (13)	0.0449 (4)	
H2A	0.2112 (15)	0.2825 (16)	0.7953 (15)	0.050 (6)*	
H4B	0.531 (2)	0.2199 (16)	0.2235 (14)	0.050 (6)*	
H4A	0.7276 (16)	0.2181 (17)	0.2166 (16)	0.058 (7)*	
H2B	0.409 (2)	0.2798 (17)	0.7850 (15)	0.055 (7)*	
H1A	0.619 (2)	0.3551 (17)	0.9244 (14)	0.051 (6)*	
H3A	0.322 (2)	0.1430 (16)	0.0815 (13)	0.045 (6)*	

### Atomic displacement parameters (Å^2^)

**Table:**

	*U*^11^	*U*^22^	*U*^33^	*U*^12^	*U*^13^	*U*^23^
C1	0.0265 (8)	0.0429 (10)	0.0285 (8)	0.0011 (7)	0.0034 (6)	0.0043 (7)
C2	0.0497 (12)	0.0530 (12)	0.0423 (11)	−0.0155 (9)	−0.0094 (9)	0.0114 (10)
C3	0.0541 (13)	0.0694 (15)	0.0389 (11)	−0.0148 (11)	−0.0147 (9)	0.0157 (10)
C4	0.0383 (10)	0.0593 (12)	0.0387 (10)	0.0060 (9)	0.0052 (8)	0.0166 (9)
C5	0.0553 (13)	0.0522 (12)	0.0472 (12)	−0.0124 (10)	0.0042 (10)	0.0140 (10)
C6	0.0486 (11)	0.0569 (13)	0.0341 (10)	−0.0154 (10)	−0.0048 (8)	0.0050 (9)
C7	0.0627 (15)	0.0858 (18)	0.0588 (15)	0.0065 (13)	0.0054 (11)	0.0386 (14)
O1	0.0392 (7)	0.0579 (9)	0.0471 (8)	−0.0029 (6)	0.0109 (6)	0.0176 (7)
O2	0.0492 (8)	0.0618 (9)	0.0292 (7)	0.0042 (7)	0.0089 (6)	0.0047 (6)
O3	0.0341 (7)	0.0696 (10)	0.0431 (8)	0.0140 (6)	0.0050 (6)	0.0164 (7)
S1	0.0285 (2)	0.0514 (3)	0.0289 (2)	0.00414 (18)	0.00579 (16)	0.00929 (19)
C14	0.0279 (8)	0.0418 (10)	0.0281 (8)	0.0000 (7)	0.0043 (6)	0.0060 (7)
C15	0.0591 (13)	0.0552 (13)	0.0343 (10)	0.0181 (10)	0.0157 (9)	0.0065 (9)
C16	0.0624 (13)	0.0512 (12)	0.0461 (12)	0.0192 (10)	0.0076 (10)	0.0129 (10)
C17	0.0374 (10)	0.0538 (12)	0.0359 (10)	−0.0053 (8)	0.0003 (8)	0.0151 (9)
C18	0.0590 (13)	0.0651 (14)	0.0342 (10)	0.0124 (11)	0.0193 (9)	0.0106 (10)
C19	0.0527 (12)	0.0499 (12)	0.0413 (11)	0.0156 (9)	0.0171 (9)	0.0111 (9)
C20	0.0603 (14)	0.0771 (17)	0.0494 (13)	−0.0044 (12)	−0.0013 (10)	0.0302 (12)
O4	0.0337 (7)	0.0652 (9)	0.0513 (8)	0.0065 (6)	−0.0015 (6)	0.0226 (7)
O5	0.0446 (7)	0.0630 (9)	0.0281 (7)	−0.0099 (6)	0.0044 (5)	0.0064 (6)
O6	0.0335 (7)	0.0574 (9)	0.0437 (7)	−0.0099 (6)	0.0020 (6)	0.0163 (6)
S2	0.0269 (2)	0.0506 (3)	0.0298 (2)	−0.00331 (18)	0.00094 (16)	0.01147 (19)
C21	0.0292 (8)	0.0340 (9)	0.0360 (9)	0.0009 (7)	0.0005 (7)	0.0140 (7)
C22	0.0264 (8)	0.0400 (10)	0.0446 (10)	0.0042 (7)	0.0037 (7)	0.0140 (8)
C23	0.0367 (9)	0.0460 (11)	0.0420 (10)	0.0081 (8)	0.0109 (8)	0.0144 (9)
C24	0.0405 (10)	0.0438 (10)	0.0328 (9)	0.0034 (8)	0.0024 (7)	0.0117 (8)
C25	0.0330 (9)	0.0334 (9)	0.0367 (9)	−0.0004 (7)	−0.0012 (7)	0.0154 (7)
C26	0.0346 (10)	0.0527 (12)	0.0464 (11)	−0.0040 (8)	−0.0051 (8)	0.0177 (9)
N3	0.0269 (7)	0.0378 (8)	0.0345 (8)	0.0008 (6)	0.0036 (6)	0.0140 (6)
N4	0.0319 (8)	0.0622 (11)	0.0351 (9)	−0.0002 (8)	0.0003 (7)	0.0098 (8)
C8	0.0309 (8)	0.0348 (9)	0.0366 (9)	0.0069 (7)	0.0038 (7)	0.0139 (7)
C9	0.0303 (9)	0.0456 (11)	0.0445 (10)	0.0088 (7)	0.0074 (7)	0.0154 (8)
C10	0.0436 (10)	0.0532 (12)	0.0445 (11)	0.0133 (9)	0.0175 (9)	0.0185 (9)
C11	0.0497 (11)	0.0494 (11)	0.0316 (9)	0.0083 (9)	0.0052 (8)	0.0133 (8)
C12	0.0379 (9)	0.0333 (9)	0.0353 (9)	0.0038 (7)	0.0016 (7)	0.0149 (7)
C13	0.0397 (10)	0.0508 (12)	0.0457 (11)	−0.0001 (9)	−0.0040 (8)	0.0167 (9)
N1	0.0301 (7)	0.0365 (8)	0.0325 (8)	0.0055 (6)	0.0055 (6)	0.0116 (6)
N2	0.0328 (8)	0.0611 (11)	0.0365 (9)	0.0045 (8)	0.0028 (7)	0.0081 (8)

### Geometric parameters (Å, º)

**Table:**

C1—C6	1.375 (3)	O6—S2	1.4497 (13)
C1—C2	1.380 (2)	C21—N4	1.325 (2)
C1—S1	1.7609 (18)	C21—N3	1.352 (2)
C2—C3	1.375 (3)	C21—C22	1.405 (3)
C2—H2	0.9300	C22—C23	1.357 (3)
C3—C4	1.372 (3)	C22—H22	0.9300
C3—H3	0.9300	C23—C24	1.401 (3)
C4—C5	1.378 (3)	C23—H23	0.9300
C4—C7	1.504 (3)	C24—C25	1.353 (3)
C5—C6	1.381 (3)	C24—H24	0.9300
C5—H5	0.9300	C25—N3	1.362 (2)
C6—H6	0.9300	C25—C26	1.493 (2)
C7—H7A	0.9600	C26—H26A	0.9600
C7—H7B	0.9600	C26—H26B	0.9600
C7—H7C	0.9600	C26—H26C	0.9600
O1—S1	1.4499 (14)	N3—H3A	0.894 (9)
O2—S1	1.4605 (14)	N4—H4B	0.876 (9)
O3—S1	1.4469 (13)	N4—H4A	0.876 (10)
C14—C15	1.375 (3)	C8—N2	1.325 (2)
C14—C19	1.378 (3)	C8—N1	1.347 (2)
C14—S2	1.7636 (18)	C8—C9	1.406 (3)
C15—C16	1.379 (3)	C9—C10	1.357 (3)
C15—H15	0.9300	C9—H9	0.9300
C16—C17	1.382 (3)	C10—C11	1.398 (3)
C16—H16	0.9300	C10—H10	0.9300
C17—C18	1.375 (3)	C11—C12	1.356 (3)
C17—C20	1.507 (3)	C11—H11	0.9300
C18—C19	1.380 (3)	C12—N1	1.360 (2)
C18—H18	0.9300	C12—C13	1.491 (3)
C19—H19	0.9300	C13—H13A	0.9600
C20—H20A	0.9600	C13—H13B	0.9600
C20—H20B	0.9600	C13—H13C	0.9600
C20—H20C	0.9600	N1—H1A	0.900 (9)
O4—S2	1.4485 (14)	N2—H2A	0.873 (10)
O5—S2	1.4582 (14)	N2—H2B	0.877 (10)
			
C6—C1—C2	119.11 (18)	O4—S2—C14	106.93 (8)
C6—C1—S1	120.76 (13)	O6—S2—C14	106.19 (8)
C2—C1—S1	120.13 (15)	O5—S2—C14	106.66 (8)
C3—C2—C1	119.76 (19)	N4—C21—N3	118.92 (16)
C3—C2—H2	120.1	N4—C21—C22	123.44 (16)
C1—C2—H2	120.1	N3—C21—C22	117.63 (16)
C4—C3—C2	122.01 (18)	C23—C22—C21	119.40 (16)
C4—C3—H3	119.0	C23—C22—H22	120.3
C2—C3—H3	119.0	C21—C22—H22	120.3
C3—C4—C5	117.70 (19)	C22—C23—C24	120.92 (17)
C3—C4—C7	120.99 (19)	C22—C23—H23	119.5
C5—C4—C7	121.3 (2)	C24—C23—H23	119.5
C4—C5—C6	121.2 (2)	C25—C24—C23	119.41 (17)
C4—C5—H5	119.4	C25—C24—H24	120.3
C6—C5—H5	119.4	C23—C24—H24	120.3
C1—C6—C5	120.25 (18)	C24—C25—N3	118.87 (16)
C1—C6—H6	119.9	C24—C25—C26	124.50 (17)
C5—C6—H6	119.9	N3—C25—C26	116.63 (16)
C4—C7—H7A	109.5	C25—C26—H26A	109.5
C4—C7—H7B	109.5	C25—C26—H26B	109.5
H7A—C7—H7B	109.5	H26A—C26—H26B	109.5
C4—C7—H7C	109.5	C25—C26—H26C	109.5
H7A—C7—H7C	109.5	H26A—C26—H26C	109.5
H7B—C7—H7C	109.5	H26B—C26—H26C	109.5
O3—S1—O1	113.06 (9)	C21—N3—C25	123.75 (15)
O3—S1—O2	111.56 (8)	C21—N3—H3A	119.2 (13)
O1—S1—O2	111.86 (8)	C25—N3—H3A	117.0 (13)
O3—S1—C1	107.19 (8)	C21—N4—H4B	121.1 (15)
O1—S1—C1	106.16 (8)	C21—N4—H4A	118.4 (16)
O2—S1—C1	106.52 (9)	H4B—N4—H4A	118 (2)
C15—C14—C19	119.43 (18)	N2—C8—N1	119.09 (16)
C15—C14—S2	120.43 (14)	N2—C8—C9	123.12 (16)
C19—C14—S2	120.03 (15)	N1—C8—C9	117.77 (16)
C14—C15—C16	120.19 (18)	C10—C9—C8	119.13 (17)
C14—C15—H15	119.9	C10—C9—H9	120.4
C16—C15—H15	119.9	C8—C9—H9	120.4
C15—C16—C17	121.08 (19)	C9—C10—C11	121.08 (18)
C15—C16—H16	119.5	C9—C10—H10	119.5
C17—C16—H16	119.5	C11—C10—H10	119.5
C18—C17—C16	117.94 (18)	C12—C11—C10	119.44 (18)
C18—C17—C20	120.96 (19)	C12—C11—H11	120.3
C16—C17—C20	121.1 (2)	C10—C11—H11	120.3
C17—C18—C19	121.60 (18)	C11—C12—N1	118.61 (17)
C17—C18—H18	119.2	C11—C12—C13	124.54 (17)
C19—C18—H18	119.2	N1—C12—C13	116.85 (16)
C14—C19—C18	119.74 (19)	C12—C13—H13A	109.5
C14—C19—H19	120.1	C12—C13—H13B	109.5
C18—C19—H19	120.1	H13A—C13—H13B	109.5
C17—C20—H20A	109.5	C12—C13—H13C	109.5
C17—C20—H20B	109.5	H13A—C13—H13C	109.5
H20A—C20—H20B	109.5	H13B—C13—H13C	109.5
C17—C20—H20C	109.5	C8—N1—C12	123.96 (15)
H20A—C20—H20C	109.5	C8—N1—H1A	118.4 (14)
H20B—C20—H20C	109.5	C12—N1—H1A	117.6 (14)
O4—S2—O6	112.84 (9)	C8—N2—H2A	115.9 (15)
O4—S2—O5	112.66 (8)	C8—N2—H2B	120.4 (15)
O6—S2—O5	111.06 (8)	H2A—N2—H2B	120 (2)
			
C6—C1—C2—C3	0.9 (3)	C15—C14—S2—O4	151.01 (16)
S1—C1—C2—C3	−178.58 (18)	C19—C14—S2—O4	−32.74 (18)
C1—C2—C3—C4	−0.5 (4)	C15—C14—S2—O6	−88.27 (17)
C2—C3—C4—C5	−0.4 (4)	C19—C14—S2—O6	87.97 (17)
C2—C3—C4—C7	−179.5 (2)	C15—C14—S2—O5	30.25 (18)
C3—C4—C5—C6	0.8 (3)	C19—C14—S2—O5	−153.50 (15)
C7—C4—C5—C6	179.9 (2)	N4—C21—C22—C23	−179.93 (18)
C2—C1—C6—C5	−0.5 (3)	N3—C21—C22—C23	1.0 (3)
S1—C1—C6—C5	178.96 (17)	C21—C22—C23—C24	−0.7 (3)
C4—C5—C6—C1	−0.3 (3)	C22—C23—C24—C25	0.0 (3)
C6—C1—S1—O3	−81.39 (18)	C23—C24—C25—N3	0.3 (3)
C2—C1—S1—O3	98.11 (17)	C23—C24—C25—C26	179.47 (17)
C6—C1—S1—O1	157.51 (16)	N4—C21—N3—C25	−179.79 (17)
C2—C1—S1—O1	−22.99 (19)	C22—C21—N3—C25	−0.7 (3)
C6—C1—S1—O2	38.15 (18)	C24—C25—N3—C21	0.0 (3)
C2—C1—S1—O2	−142.35 (17)	C26—C25—N3—C21	−179.21 (16)
C19—C14—C15—C16	−0.9 (3)	N2—C8—C9—C10	−179.67 (19)
S2—C14—C15—C16	175.40 (17)	N1—C8—C9—C10	−1.1 (3)
C14—C15—C16—C17	−0.3 (3)	C8—C9—C10—C11	0.6 (3)
C15—C16—C17—C18	1.0 (3)	C9—C10—C11—C12	−0.3 (3)
C15—C16—C17—C20	−178.9 (2)	C10—C11—C12—N1	0.4 (3)
C16—C17—C18—C19	−0.6 (3)	C10—C11—C12—C13	−178.58 (18)
C20—C17—C18—C19	179.3 (2)	N2—C8—N1—C12	179.90 (17)
C15—C14—C19—C18	1.2 (3)	C9—C8—N1—C12	1.3 (3)
S2—C14—C19—C18	−175.05 (16)	C11—C12—N1—C8	−0.9 (3)
C17—C18—C19—C14	−0.5 (3)	C13—C12—N1—C8	178.12 (16)

### Hydrogen-bond geometry (Å, º)

**Table:**

*D*—H···*A*	*D*—H	H···*A*	*D*···*A*	*D*—H···*A*
N1—H1*A*···O2	0.90 (1)	1.88 (1)	2.772 (2)	171 (2)
N2—H2*A*···O3^i^	0.87 (1)	2.01 (1)	2.880 (2)	174 (2)
N2—H2*B*···O1	0.88 (1)	2.07 (1)	2.919 (2)	162 (2)
N3—H3*A*···O5	0.89 (1)	1.90 (1)	2.789 (2)	174 (2)
N4—H4*A*···O4^ii^	0.88 (1)	2.02 (1)	2.882 (2)	167 (2)
N4—H4*B*···O6	0.88 (1)	2.04 (1)	2.883 (2)	162 (2)
C22—H22···O5^ii^	0.93	2.58	3.455 (2)	157

Symmetry codes: (i) *x*−1, *y*, *z*; (ii) *x*+1, *y*, *z*.
